# Spectroelectrochemistry of Water Oxidation Kinetics in Molecular versus Heterogeneous Oxide Iridium Electrocatalysts

**DOI:** 10.1021/jacs.2c02006

**Published:** 2022-05-05

**Authors:** Carlota Bozal-Ginesta, Reshma R. Rao, Camilo A. Mesa, Yuanxing Wang, Yanyan Zhao, Gongfang Hu, Daniel Antón-García, Ifan E. L. Stephens, Erwin Reisner, Gary W. Brudvig, Dunwei Wang, James R. Durrant

**Affiliations:** † Department of Chemistry, Centre for Processable Electronics, Imperial College London, 80 Wood Lane, London W12 0BZ, U.K.; ‡ Department of Chemistry, Boston College, 2609 Beacon Street, Chestnut Hill, Massachusetts 02467, United States; § Yale Energy Sciences Institute and Department of Chemistry, Yale University, New Haven, Connecticut 06520, United States; ∥ Yusuf Hamied Department of Chemistry, University of Cambridge, Lensfield Road, Cambridge CB2 1EW, U.K.; ⊥ Department of Materials, Imperial College London, 80 Wood Lane, London W12 0BZ, U.K.

## Abstract

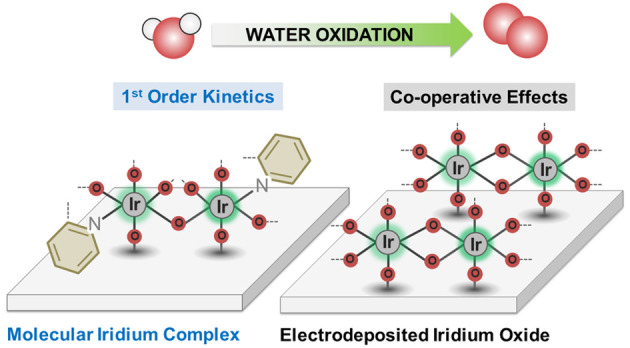

Water oxidation is the step limiting the efficiency of electrocatalytic hydrogen production from water. Spectroelectrochemical analyses are employed to make a direct comparison of water oxidation reaction kinetics between a molecular catalyst, the dimeric iridium catalyst [Ir_2_(pyalc)_2_(H_2_O)_4_-(μ-O)]^2+^ (**Ir**_**Molecular**_, pyalc = 2-(2′pyridinyl)-2-propanolate) immobilized on a mesoporous indium tin oxide (ITO) substrate, with that of an heterogeneous electrocatalyst, an amorphous hydrous iridium (**IrO**_*x*_) film. For both systems, four analogous redox states were detected, with the formation of Ir(4+)–Ir(5+) being the potential-determining step in both cases. However, the two systems exhibit distinct water oxidation reaction kinetics, with potential-independent first-order kinetics for **Ir**_**Molecular**_ contrasting with potential-dependent kinetics for **IrO**_*x*_. This is attributed to water oxidation on the heterogeneous catalyst requiring co-operative effects between neighboring oxidized Ir centers. The ability of **Ir**_**Molecular**_ to drive water oxidation without such co-operative effects is explained by the specific coordination environment around its Ir centers. These distinctions between molecular and heterogeneous reaction kinetics are shown to explain the differences observed in their water oxidation electrocatalytic performance under different potential conditions.

Water oxidation catalysis is a key challenge for hydrogen synthesis from water via electrolysis, with extensive interest in both heterogeneous oxides and molecular catalysts.^1−3^ While heterogeneous oxides are already employed in commercial electrolyzers, there have been striking recent advances in the performance and stability of molecular water oxidation catalysts based on iridium.^4−7^ Molecular catalysts are particularly attractive as model systems for this reaction, with their well-defined and tunable atomic structures aiding mechanistic insight.^7−10^ A key potential mechanistic difference is that molecular catalysts typically consist of isolated metal centers, while heterogeneous oxide films are often reported to promote water oxidation by co-operative effects between adjacent sites.^11−13^ Recent work has controversially suggested that long-range interactions, which depend on the coverage of oxidized *O species, can be attributed to solvation effects on IrO_*x*_ surfaces.^11^ However, direct comparisons between the water oxidation kinetics of molecular and heterogeneous catalysts operating under similar reaction conditions have been very limited to date.^14,15^ Herein we use *operando* transient spectroelectrochemistry to compare two highly active water-oxidation electrocatalysts based on iridium: a dimeric molecular catalyst [Ir_2_(pyalc)_2_(H_2_O)_4_-(μ-O)]^2+^ (**Ir**_**Molecular**_, Figure 1A) (pyalc = 2-(2′pyridinyl)-2-propanolate) and the amorphous hydrous iridium oxide (**IrO**_**x,**_Figure 1B).^2,3,7,16−20^

**Figure 1:**
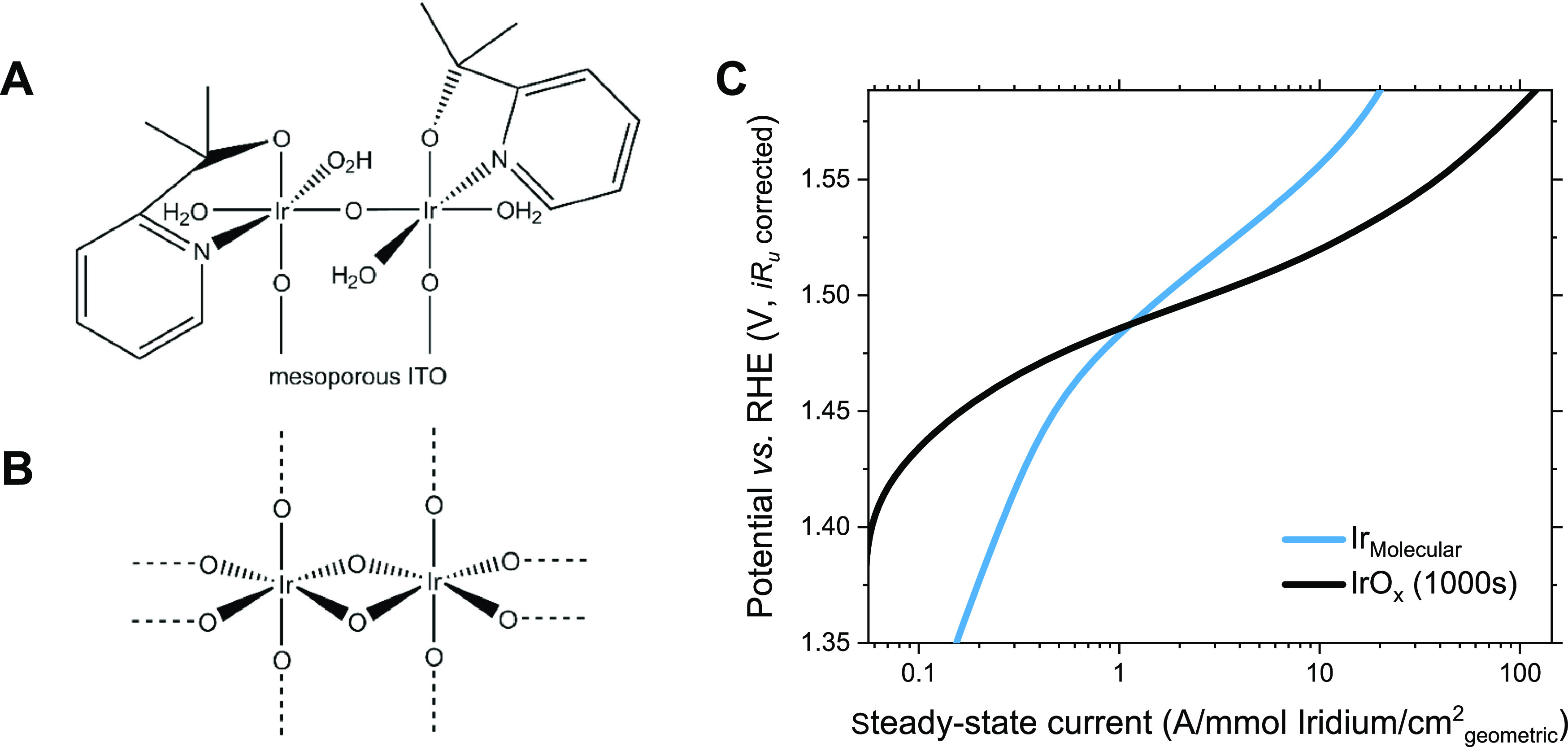
(A) Proposed molecular structure of **Ir**_**Molecula**r_ and (B) schematic of a cluster in electrodeposited **IrO**_*x*_, proposed by Pavlovic et al.^13,20,21^ (C) Tafel plot from the steady-state current per mol of electrochemically active iridium every 50 mV of **Ir**_**Molecular**_ and **IrO**_*x*_ (electrodeposited for 1000s) in aqueous HClO_4_ 0.1 M at pH 1.2. The calculation of electrochemically active iridium is described in the Supporting Information.

It has previously been reported that molecular iridium catalysts can be immobilized on oxide surfaces without the need for additional anchoring groups. When immobilized on high-surface area mesoporous indium tin oxide (mesoITO), **Ir**_**Molecular**_ can reach turnover frequencies around 7.9 s^–1^ at an overpotential of 520 mV, and its ligands remain intact for at least 11 h at pH 2.6 under 1.55 V_RHE_.^16^**Ir**_**Molecular**_ cocatalysts have also been reported to enhance the performance of hematite photoanodes, shifting the oxygen evolution reaction (OER) onset potential by ∼250 mV.^14,15,21^ In our previous work on **IrO**_*x*_ electrodes, spectroelectrochemical techniques were applied to identify three redox transitions, attributed following the literature to the oxidation of Ir^3+^ to Ir^3.x+^, Ir^3.x+^ to Ir^4+^, and Ir^4+^ to Ir^4.y+^, where Ir^3.x+^ and Ir^4.y+^ represent mixed valence states including Ir^3+^ and Ir^4+^, and oxidized Ir^4+^ respectively.^22−39^ The final redox transition results in the formation of oxygenated species, *O, whose OER kinetics can also be probed using time-resolved spectroelectrochemistry.^22^ Herein, we extend this spectroelectrochemical analysis to the dimeric complex **Ir**_**Molecular**_ immobilized on mesoITO. By comparing the molecular redox states and their kinetics to those of electrodeposited **IrO**_*x*_ films,^22^ we aim to understand the impact on OER activity of the specific coordination environment of iridium centers in **Ir**_**Molecular**_ as well as the potential impact of co-operative effects between Ir centers present in the heterogeneous **IrO**_*x*_ film.

The molecular iridium catalyst **Ir**_**Molecular**_ was immobilized on mesoporous ITO (mesoITO) following a previously reported procedure, forming a packed monolayer of ∼0.04 μmol_Ir_/cm^2^_geometric_, while **IrO**_*x*_ films were electrodeposited in water from an iridium salt.^14−16^ Iridium centers in mesoITO-**Ir**_**Molecular**_ are expected to be at least 3 times further away from the iridium centers in adjacent molecules than between iridium centers in one **Ir**_**Molecular**_ catalyst and in **IrO**_*x*_ because of the bulkiness of the ligands.^14,16^ The Tafel plot of the resulting **Ir**_**Molecular**_ films (Figure 1C) shows an exponential increase in the^26,27^ current at a potential of ∼1.32 V_RHE_, ∼80 mV lower than in **IrO**_*x*_. Comparing the catalytic waves for water oxidation in Figure 1C, the **Ir**_**Molecular**_ films exhibit higher performance below ∼1.45 V_RHE_ with a Tafel slope of 174 mV/dec. Above 1.45 V_RHE_, **IrO**_*x*_ films exhibit a sharper onset and thus higher performance than **Ir**_**Molecular**_, with Tafel slopes of 59 and 69 mV/dec, respectively, in accordance with previous studies.^11,16^

The redox chemistry of **Ir**_**Molecular**_ on mesoITO was analyzed further spectroelectrochemically, following procedures we have previously reported for **IrO**_*x*_ (Figures S2–S3).^22^ Its UV–vis absorption spectrum was measured as a function of applied potential with 5 mV increments in aqueous 0.1 M HClO_4_ electrolyte at pH 1.2 (Figure 2A). For the potential range 0.78–1.08 V_RHE_, an absorption band at 590 nm dominated the spectrum. A new feature appeared at 800 nm between 1.13 and 1.43 V_RHE_, and above 1.43 V_RHE_, absorption changes were primarily detected below 500 nm. These features were deconvolved (Figures 2B and S3–S7) by fitting a model which assumes three additive contributions to the absorption, each linearly proportional to the concentration of redox states, and where the concentration of redox states formed at each potential follows a Gaussian distribution (SI, Equations S2–7), as reported in our previous work.^22^ The deconvoluted spectroelectrochemistry results are shown in Figure 2B, where the normalized concentration distributions and differential absorption corresponding to the three redox transitions detected in **Ir**_**Molecular**_ are compared to those in **IrO**_*x*_. The optical signals related to the three redox transitions in the two catalysts have broadly similar features (Figure 2B left). However, the absorption bands are slightly blue-shifted in **Ir**_**Molecular**_ (shown in more detail in Figure S8). The concentration distributions in **Ir**_**Molecular**_ are shifted anodically by ∼100 mV with respect to **IrO**_*x*_ for all the three redox transitions (Figure 2B right).

**Figure 2:**
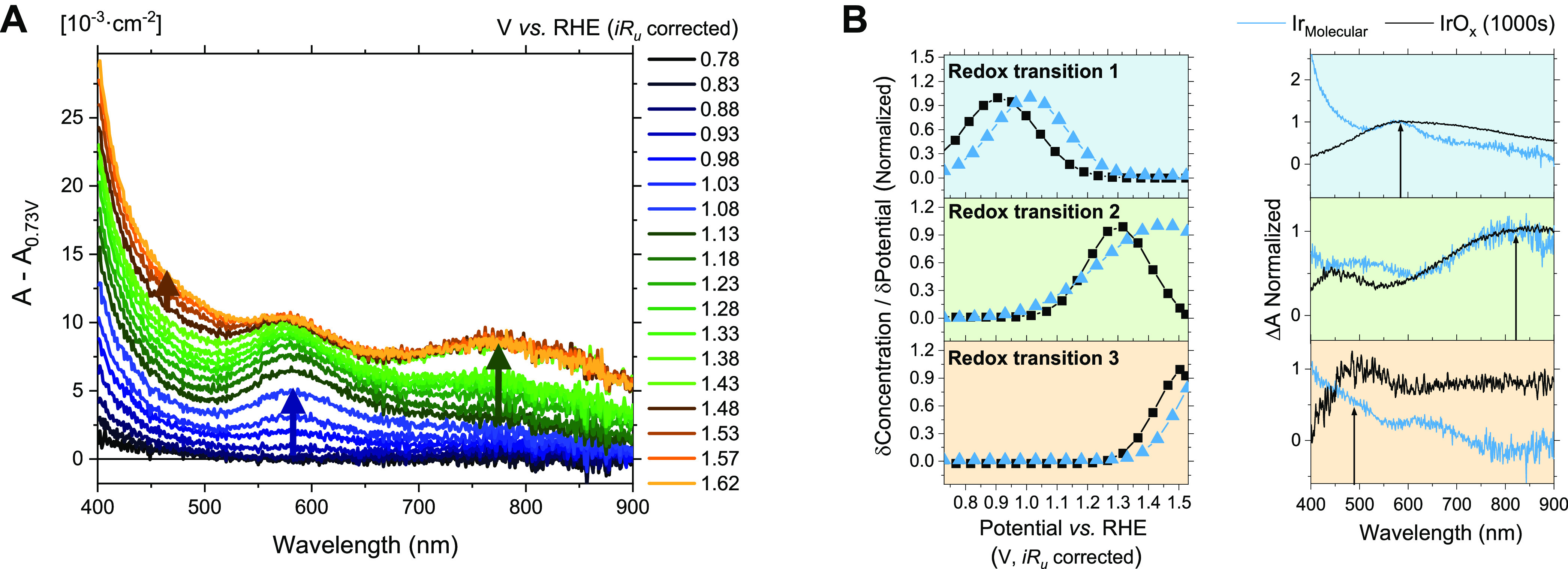
(A) Absorption changes at different potentials of **Ir**_**Molecular**_ on mesoporous ITO. (B) Normalized deconvolution results of the spectroelectrochemical data of **Ir**_**Molecular**_ and **IrO**_*x*_: (*left*) change in the concentration of the redox states formed at increasing potentials and (*right*) differential absorption coefficients of the corresponding redox transitions (relative to 0.73 V vs RHE). Measurements done in aqueous HClO_4_ 0.1 M at pH 1.2 under constant applied potentials every 0.05 V.

Considering the assignments of the redox transitions in Figure 2 for **IrO**_*x*_, the transitions in the 0.8–1.4 V_RHE_ range have been assigned to intervalence charge transfer within the iridium d orbitals derived from the oxidation of Ir(3+) to Ir(4+) and deprotonation of hydroxyl groups coordinated to the Ir center.^23,24,32,35,40−42^ Positive of 1.4 V_RHE_, redox states of iridium higher than 4+ are expected to be formed.^29,33,34,43−45^ Crabtree et al. have reported similar redox transitions in an iridium dimer structurally analogous to **Ir**_**Molecular**_: Ir(3+)–Ir(3+) absorbing below 450 nm, Ir(4+)–Ir(4+) absorbing at 600–750 nm, and Ir(4+)–Ir(5+) absorbing at 500 nm.^46,47^ By taking this iridium dimer as a reference, the three redox transitions we observe in **Ir**_**Molecular**_ can be assigned to the sequential oxidation of Ir(3+)–Ir(3+) to Ir(3+)–Ir(4+), Ir(4+)–Ir(4+), and Ir(4+)–Ir(5+), respectively, in good agreement with assignments on **IrO**_*x*_. The absolute absorption and the calculated concentrations are smaller for mesoITO-**Ir**_**Molecular**_ compared to electrodeposited **IrO**_*x*_ (Figures 2A and S7 for **Ir**_**Molecular**_ and S1B and S2 for **IrO**_*x*_), indicative of larger geometric densities of electrochemically active Ir centers in **IrO**_*x*_, and attributed to its permeability to the electrolyte and electrode morphology. By comparing the electrochemical and the deconvoluted spectroelectrochemical data in Figures 1C and 2B, respectively, it is apparent for both **Ir**_**Molecular**_ and **IrO**_*x*_ that the electrocatalytic current overlaps with the third redox transition detected spectroelectrochemically (discussed further below). This indicates that this transition, assigned to Ir(4+)–Ir(5+) formation in **Ir**_**Molecular**_, enables O_2_ evolution, similar to our findings on **IrO**_*x*_. It is also apparent that this transition is shifted anodically for the **Ir**_**Molecular**_ relative to **IrO**_*x*_, indicative of the role of ligands in tuning the redox activity of the Ir centers.

To analyze the kinetics of the redox states in **Ir**_**Molecular**_, the optical signal decay was measured after turning an applied potential off, following the same procedure reported for **IrO**_*x*_ in our previous study (Scheme S1 and Supporting Information).^22^ This methodology was used to deduce the OER reaction kinetics of the active redox state as a function of its concentration. Absorption changes upon applying an electric potential from the open circuit potential (OCP) were detected only at applied potentials above ∼1.32 V_RHE_ (*iR*_*u*_ corrected) (Figure 3A), which corresponds to the potential at which the active redox state Ir(4+)–Ir(5+) is formed. Notably, the decay kinetics of the active state optical signal in **Ir**_**Molecular**_ are almost invariant throughout the 1.3–1.6 V_RHE_ range, implying a potential-independent first-order mechanism, where the rate of the reaction (*J*) is proportional to a potential-independent rate constant (*k*) and the concentration of oxidized species (θ); i.e., *J = kθ*. This contrasts with the behavior of **IrO**_*x*_, where the decay of the optical signal becomes substantially faster with increasing potentials in this range. The corresponding decay time constants are plotted in Figure 3B, where the optical signal lifetimes at different potentials were extracted from fitting the signal decays with an initial linear regression (Equations S10–S11). It is apparent that, at potentials below 1.45 V_RHE_, the lifetimes of the active state in **IrO**_*x*_ are longer than in **Ir**_**Molecular**_, indicative of lower reactivity, while above 1.45 V_RHE_ the lifetimes become shorter, indicative of higher reactivity.

**Figure 3:**
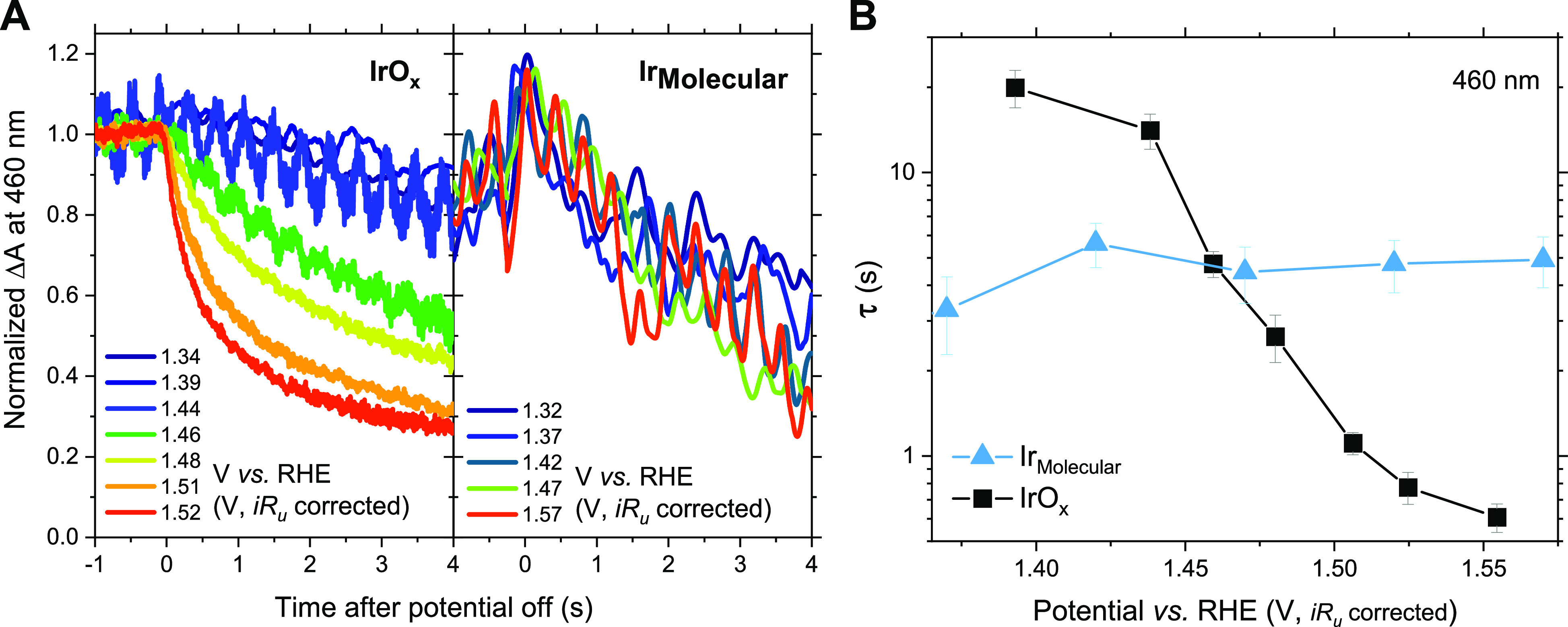
(A) Absorption decay after turning the potential off, and (B) optical signal lifetimes derived from fitting an initial linear regression in Figure 3A in **Ir**_**Molecular**_ and **IrO**_*x*_ in 0.1 M HClO_4_ water at pH 1.2.

Apart from the shift in oxidation potential discussed above, **IrO**_*x*_ and **Ir**_**Molecular**_ show similar potential dependencies for the third redox transition assigned to the formation of the oxidized species driving OER (Figure 2B). As such, the smaller Tafel slope observed for **IrO**_*x*_ (Figure 2A) can be assigned to a sharper acceleration of OER reaction kinetics for this film with potential. The absence of any potential dependence of reaction kinetics for **Ir**_**Molecular**_ suggests that this acceleration for **IrO**_*x*_ is unlikely to be due to a mechanism change or potential-dependent change in reaction activation energy.

For **Ir**_**Molecular**_ the third redox transition (Figure 2B) coincides with the potential required to reach 0.1 A/mmol/cm^2^_geometric_ (Figure 1C), consistent with its first-order OER kinetics. In contrast, for **IrO**_*x*_ the potential to reach 0.1 A/mmol/cm^2^_geometric_ is shifted anodically by ∼100 mV relative to its third redox transition, providing further confirmation that this heterogeneous catalyst needs to reach a critical concentration of redox active state to trigger the OER reaction. Therefore, it appears likely that this acceleration results from co-operative effects between different oxidized Ir centers on **IrO**_*x*_,^48^ consistent with a recent report by Nong et al.^11^ of co-operative interactions between multiple sites on IrO_*x*_ during OER. Such co-operative effects cannot be realized in the isolated Ir centers of mesoITO-**Ir**_**Molecular**_ due to steric hindrance by the ligands.

At low overpotentials, in the absence of co-operative effects that can enhance OER activity on **IrO**_**x**_, the reactivity for both catalysts would be governed by the chemical environment of isolated oxidized Ir centers, including the ligands and the electrode substrate, as illustrated in Scheme 1. In the molecular catalyst, the absorption maxima above 500 nm are slightly blue-shifted (Figure 2B right), and the potential for each redox transition in **Ir**_**Molecular**_ is shifted by ∼100 mV compared to **IrO**_*x*_ (Figure 2B left). Considering that the Ir centers have an octahedral configuration in both **Ir**_**Molecular**_ and **IrO**_*x*_, a plausible cause for these shifts is different solvation environment in **Ir**_**Molecular**_ and **IrO**_*x*_ and the stabilization of the *t*_2*g*_ d-based iridium molecular orbitals in **Ir**_**Molecular**_ by pyridine π orbitals (Scheme 1).^49,50^ On the one hand, this would lead to a larger energy gap between the *t*_2*g*_ and *e*_*g*_ molecular orbitals, which corresponds to the transition observed in the visible range.^49,50^ On the other hand, this stabilization of the *t*_2*g*_ molecular orbitals, where the valence electrons are located, would make the iridium centers in **Ir**_**Molecular**_ harder to oxidize than in **IrO**_*x*_. This is in agreement with the seminal work of Rossmeisl and Nørskov et al.^51^ which suggested that IrO_2_(110) binds oxygen too strongly relative to the optimal catalyst, resulting in the O–O bond formation step to form *OOH being rate-determining. Consequently, the faster reaction kinetics, and higher OER activity of **Ir**_**Molecular**_ at low overpotentials (<1.45 V_RHE_), can thus be explained by the stabilization effect of the pyridine ligand on the valence iridium d orbitals, which increases the oxidizing potential of Ir and decreases the oxygen binding strength.

**Scheme 1:**
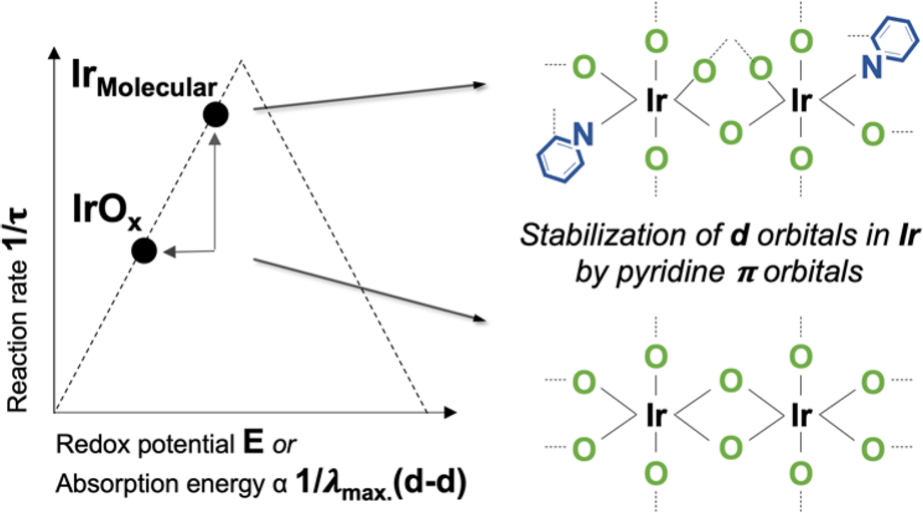
Proposed Chemical Effects of the Atoms Coordinated to the Iridium Active Sites on the Redox Potential (*E*) and Maximum Absorption Wavelength of d–d Transitions (*λ*_max._(d–d)), and lifetimes (τ) in water 0.1 M HClO_4_ at pH 1.2

In conclusion, by employing *operando* spectroelectrochemistry, we have analyzed the redox transitions and OER reaction kinetics for **IrO**_*x*_ and for **Ir**_**Molecular**_ immobilized on meso-ITO between 1 and 1.6 V_RHE_. The third redox transition, assigned in both systems to Ir(4+) oxidation, was observed to correlate with the increase in the OER current. **Ir**_**Molecular**_ is observed to exhibit potential independent OER reaction kinetics, indicative of water oxidation by independent molecular catalysts. This contrasts with **IrO**_*x*_ films, where the reaction kinetics are observed to accelerate strongly with applied potential, attributed to the OER on this catalyst requiring co-operative interactions between neighboring oxidized Ir centers. The ability of **Ir**_**Molecular**_ to drive OER without requiring such co-operative effects is attributed to the anodic shift of its redox transitions relative to **IrO**_*x*_, resulting from the specific chemical environment of its iridium centers. These differences in redox state energetics and OER kinetics explain the differences observed in OER activity and Tafel slopes between these molecular and heterogeneous catalysts. The absence of co-operative effects, as observed herein for our molecular system, could limit the performance of single atom catalysts^52−55^ for OER, particularly on Ir-based centers. Our study has therefore allowed a direct comparison of molecular catalysts and heterogeneous oxide film OER kinetics, thus providing insights into how both the local environment of the catalytic site and co-operative effects between oxidized sites can result in significantly different water oxidation kinetics.

## Supporting Information Available

Preparation and measurement procedures, Scheme S1, Equations S1–S9, Figures S1–S14, and Table S1. All the raw data are available on Zenodo (10.5281/zenodo.6407221). (PDF)
