# Supplementary material for: Spectroelectrochemistry of Water Oxidation Kinetics in Molecular versus Heterogeneous Oxide Iridium Electrocatalysts
Source: J Am Chem Soc. 2022 May 5;144(19):8454–9. doi: 10.1021/jacs.2c02006 (PMC9121376; doi:10.1021/jacs.2c02006)
Supplement: Supplementary file 1 — ja2c02006_si_001.pdf [file ja2c02006_si_001.pdf]

Supporting Information

Spectroelectrochemistry of water oxidation kinetics in molecular *versus* heterogeneous oxide iridium electrocatalysts

Carlota Bozal-Ginesta[a†], Reshma R. Rao[a], Camilo A. Mesa[a#], Yuanxing Wang[b], Yanyan Zhao[b], Gongfang Hu[c], Daniel Antón-García[d], Ifan E.L. Stephens[e], Erwin Reisner[d], Gary W. Brudvig[c], Dunwei Wang[b] and James R. Durrant[a*]

[a]Department of Chemistry, Centre for Processable Electronics, Imperial College London, 80 Wood Lane, London W12 0BZ, UK; [b]Department of Chemistry, Boston College, 2609 Beacon St., Chestnut Hill, Massachusetts 02467, United States; [c]Yale Energy Sciences Institute and Department of Chemistry, Yale University, New Haven, Connecticut 06520, United States; [d]Yusuf Hamied Department of Chemistry, University of Cambridge, Lensfield Road, Cambridge CB2 1EW, UK; [e]Department of Materials, Imperial College London, 80 Wood Lane, London W12 0BZ, UK

Present addresses:
[†] C.B.G.: Departments of Chemistry and Computer Science, University of Toronto, Lash Miller Chemical Laboratories, 80 St George Street, M5S3H6 Toronto, Canada
[#]C.A.M.: Institute of Advanced Materials (INAM), Universitat Jaume I, Avenida de Vicent Sos Baynat, s/n, 12006 Castelló de la Plana, Spain

All the chemicals used were purchased from Sigma Aldrich, unless otherwise stated.

Preparation of mesoporous indium tin oxide films

Mesoporous indium tin oxide (ITO) films (mesoITO) with a geometrical area of ~1 cm^2 and a thickness of ~3 µm were prepared by spin-coating a dispersion of commercial ITO nanoparticles (<50 nm) onto a fluorine-doped tin oxide (FTO) substrate on glass. To clean the FTO-glass substrate, a solution with 50:17:33 v/v of HCl (37%), NH_4OH (30% NH_3 aqueous) and H_2O_2 was first prepared. The FTO-glass was soaked in the solution at 80ºC for 15 minutes, rinsed with water, consecutively sonicated in ethanol and acetone for 15 minutes each, and finally dried at 70ºC in air. The dispersion of ITO nanoparticles (20% weight) was prepared in 5 M acetic acid ethanol and sonicated for 30 minutes in an ice-cooled sonication bath. The dispersion was then spin-coated onto the clean FTO-glass, sintered at 450ºC for 30 minutes, and dried in air.

$Ir_{Molecular}$ preparation and immobilisation on mesoITO

Following a previously reported procedure,[1–3] a solution of 1.25 mM $[Ir(pyalc)(H_2O)_2\text{-}(\mu\text{-}O)]_2^{2+}$ (**$Ir_{Molecular}$**) (pyalc = 2-(2'pyridyl)-2-propanolate) was prepared by mixing 2.5 mM aqueous [Cp*Ir(pyalc)OH] (Cp = pentamethyl-cyclopentadienyl) and $NaIO_4$ with an approximate molar ratio of 1:100. The pH of the solution was then adjusted to ~3 with nitric acid. The organometallic precursor [Cp*Ir(pyalc)OH] was synthesised by following the same method reported by Hintermair *et al.*[4,5] To immobilise **$Ir_{Molecular}$** on mesoITO, the mesoITO films were soaked in the previsous **$Ir_{Molecular}$** solution for 16 h and rinsed with water.

Electrodeposition of IrO_x

The IrO_x films were all prepared from a solution of iridium salt consisting of 0.2 mmol of $Ir^{3+}Cl_3$ hydrate (*Fluorochem*) and 2 mmol of oxalic acid dehydrate in 30 mL of water. The pH of the iridium salt solution was adjusted to 10 by adding ~5mmol of Na_2CO_3 (*ReagentPlus® ≥ 99.0%*), turning the colour of the solution from yellow to green. The volume of the solution was increased to 50 mL by adding more water. The solution was left to rest for 4 days at 35ºC and then stored in the freezer at 4ºC. The electrodeposition of IrO_x from this iridium solution was done by soaking a clean FTO on a glass substrate (~1·2·0.2 cm^3) and applying a current of 35 μA. To prepare IrO_x samples with different thicknesses, the current was applied for 1000 s, 700 s, 500 s, 120 s and 60 s. Polyimide tape was attached on the FTO surface to limit the surface of the IrO_x to ~1 cm^2. This preparation procedure is similar to that in the literature.[6,7]

Spectroelectrochemistry

Spectroelectrochemistry measurements consisted in probing the absorbance of a sample with an *Agilent Technologies Cary 60* UV-Vis spectrometer under different applied potential. The potential was controlled with a *Metrohm Autolab PGSTAT101* potentiostat and applied between a platinum mesh (i.e. the counter electrode) and the sample (i.e. the working electrode). The potential at the working electrode was measured with respect to an Ag/AgCl reference electrode saturated with KCl. The absorption was recorded after applying the corresponding potential for ~5 minutes until the current was stable. Each spectrum was measured at least two times until it reached a steady state. The same results were also obtained using an alternative home-built setup integrated by an *OceanOptics HL-2000-FHSA* halogen light source and a *OceanOptics Maya2000Pro* spectrometer.

All the potentials are reported *versus* RHE and were iR_u corrected. The potentials *versus* Ag/AgCl saturated with KCl ($E_{Ag/AgCl}$) were converted into potentials *versus* RHE (E_{RHE}) as follows:

Equation S1
$$E_{RHE} = E_{Ag/AgCl} + E^0_{Ag/AgCl} + \frac{2.30 \cdot R \cdot T}{z \cdot F} \cdot pH$$
$$= E_{Ag/AgCl} + 0.197 + 0.059 \cdot pH = E_{Ag/AgCl} + 0.2678$$

Where R is the ideal gas constant (8.314 J·mol^{-1}·K^{-1}), T is the temperature (298 K), F is the Faraday constant (96485 C/mol electrons), z is the number of electrons transferred (1 mol electrons), and $E_{Ag/AgCl}{}^0$ is the standard potential of the Ag/AgCl reference electrode saturated with KCl. The pH used is 1.2.

All the potentials were corrected by subtracting $i{\cdot}R_u$, where i is the current measured at the corresponding potential and R_u is the uncompensated resistance The resistance R_u (between 35 and 40 Ω) was calculated by fitting electrochemical impedance data in the 0.1-1 Hz range with the Randles circuit model.

Step-Potential Spectroelectrochemistry

Step-potential spectroelectrochemistry measurements (SP-SEC) consisted in probing the absorption during potential cycles of two steps, as represented in Scheme S1. The potential was controlled with a *PalmSens3* potentiostat. In parallel, the probe light was produced with a 100 W *Bentham* tungsten lamp, and its wavelength was selected with two *Horiba Scientific OBB* monochromators placed before and after the sample. Additionally, a long-pass filter regulated by a mechanical colour wheel (*FW101C Thorlabs*) stood between the sample and the second monochromator. The probe light was detected by a silicon PIN photodiode (*Hamamatsu S3071*) and filtered by an optical transient amplifier (*Costronics 2004*). Finally, both the electrical and optical signals were processed with a digital phosphor oscilloscope (*Tektronix DPO 3012*) and a DAQ (*National Instruments X Series Multifunction*). In the SP-SEC measurements, the electrochemical cell and electrodes used were the same as in the spectroelectrochemical experiments above. The oscillations observed in the optical signals (~2s^{-1} Hz) are considered environmental noise.

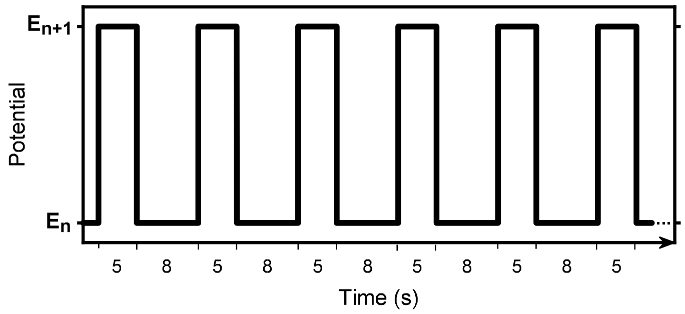

Scheme S1. Potential steps in a step-potential spectroelectrochemistry measurement (SP-SEC). Consecutively and in cycles, the potential E_n is applied during 8 s, and the potential E_{n+1} is applied during 5 s. Simultaneously, the absorption difference ΔA of the sample is monitored ($\Delta A=A(E_{n+1})-A(E_n)$).

The measurements in Figure 3 were performed by applying a potential for 5 s and then switching to open circuit for 8 seconds (zero current) following Scheme S1. During the 8-s period, the open circuit potential of the electrode changes until it plateaus at a constant value.

Data analysis and deconvolution

Spectroelectrochemical deconvolution

Spectroelectrochemical model data $\Delta A_{fit}(E,\lambda)$ was generated in *Matlab R2019a* with Equations S1-6, where ΔA is the absorption difference as a function of the potential E and the wavelength λ. The model data was built by making for major assumptions. First, the absorption changes were calculated as a sum of independent contributions $A_i(E,\lambda)$ (Equation S2), where i is the redox transition number. One, two and three absorption contributions were considered in the deconvolution of each data set. Second, the absorption changes were considered linearly proportional to the concentration of the redox state formed at each transition following the Lambert-Beer law (Equation S3), $C_i(E)$. Third, the concentration of the redox state formed at each potential was approximated as a Gaussian distribution over potential (Equation S4), expected to be a good representation of capacitive redox transitions but a less accurate approximation for the onset of catalytic and Faradaic processes if the catalytic depletion of redox states

is faster than its electrochemical recovery. Lorentzian distributions were tested to model the concentration changes over potential, but Gaussian distributions were a better fit in all the cases. Fourth, the differential absorption coefficients $\varepsilon_i(\lambda)$ were calculated from the experimental absorption at three different potentials following Equations S5-7. Positive differential absorption coefficients are expected when the resulting redox state is more absorptive than the starting redox state, and negative differential absorption coefficients are expected in the opposite case.

The model data was fit to the experimental data by adjusting the center (μ), width (σ) and amplitude (A) of the Gaussian distributions (A·*Gaussian(μ,σ)*) corresponding to the concentration changes over potential of each redox state. The model data was optimised in *Matlab* with the global minimisation tool *GlobalSearch* and the solver *fmincon*. These tools were set to find the minimum difference between the model and the real data ($|\Delta A_{real}(E,\lambda) - \Delta A_{fit}(E,\lambda)|$) across all the wavelengths and potentials. Starting with a set of 200 initial trial points, a total of 1000 trial points were examined in each data set, where each point consisted of a different combination of values for the variables A, μ and σ (Equation S4).

Equation S2 $\Delta A_{fit}(E,\lambda) = A_1(E,\lambda) + A_2(E,\lambda) + A_3(E,\lambda)$

Equation S3 $A_i(E,\lambda) = C_i(E) \cdot \varepsilon_i(\lambda)$

Equation S4 $C_i(E) = \int_{0.55}^{E} \left(\frac{\mathrm{d}C}{\mathrm{d}E}\right) \mathrm{d}E = \int_{0.55}^{E} A \cdot Gaussian(\mu,\sigma)\, \mathrm{d}E$

Equation S5 $\varepsilon_1(\lambda) = \frac{\Delta A_{real}(0.8V,\lambda)}{C_1(0.8V)}$

Equation S6 $\varepsilon_2(\lambda) = \frac{\Delta A_{real}(1V,\lambda) - C_1(1V) \cdot \varepsilon_1(\lambda)}{C_2(1V)}$

Equation S7 $\varepsilon_3(\lambda) = \frac{\Delta A_{real}(1.5V,\lambda) - C_1(1.5V) \cdot \varepsilon_1(\lambda) - C_2(1.5V) \cdot \varepsilon_2(\lambda)}{C_3(1.5V)}$

The gaussian function in Equation S4 is shown in Equation S8 below:

Equation S8 (Gaussian) $y = A \cdot e^{-\frac{1}{2}\left(\frac{x-\mu}{\sigma}\right)^2}$

Where x and y are the independent variables consisting on the potential and concentration respectively herein, and A, $\boldsymbol{\mu}$, and $\boldsymbol{\sigma}$ are the fit constants which are adjusted to the experimental results. The latter variables can be correlated with the Nernst equation (Equation S9) and the electrolyte access to the electrochemical active sites:

Equation S9 (Nernst) $[Ox] = [Red]e^{-\frac{ze(E^O - E)}{RT}}$

Where *[Ox]* and *[Red]* are the concentration of oxidized and reduced species respectively, *z* is the number of electrons exchanged, *T* is the temperature, *R* is the ideal gas constant, and E^0 and *E* are the standard reduction potential and the reduction potential respectively.

Calibration and calculation of electrochemical active sites

The concentration changes and differential absorption coefficients of the redox states that best fit the spectrelectrochemical data were adjusted with the experimental values of $\varepsilon_1(600\ nm)$, $\varepsilon_2(800\ nm)$ and $\varepsilon_3(460\ nm)$, the differential absorption coefficients at the absorption maxima during the 1st, 2nd and 3rd redox transitions respectively. To calculate these differential absorption coefficients, the current and absorption changes were simultaneously measured during step potential spectroelectrochemistry measurements (SP-SEC), where two potentials with a difference of 50 mV were cyclically applied in consecutive steps of 5 s and 8 s (Scheme S1). When changing the applied potential, the current peaks, in parallel to a change in the absorption (Figure S5). The current spike is assumed to be mostly due to the oxidation and reduction of states in the catalyst, and the absorption change is assigned to the differential absorption of the newly formed redox state relative to the starting state. In order to estimate the moles of oxidized species formed at each potential interval, we integrated the current spike over time, having subtracted the background current. For the redox transitions 1 and 2, the maximum absorptions changes at 600 nm and 800 nm were plotted against the extracted charges at potential intervals around 0.8 V and 1.3 V *vs.* RHE respectively, where the corresponding redox transition is the dominant process (Figure S5). For the redox transition 3, the same methodology was applied using the absorption changes at 460 nm and the charges extracted above 1.2 V but, because it overlaps significantly with the redox transition 2, the charges and absorption corresponding to the latter redox transition were previously subtracted. The differential absorption coefficient corresponding to a one-electron oxidation per redox transition was taken from the slope of the linear regression (Figure S6).

The electrochemically active iridium in both catalysts is considered all the iridium detected by spectroelectrochemistry. This is because the UV−vis of these compounds is related to proton-coupled electron-transfer processes that must involve iridium placed either at the electrode−electrolyte interface or, given the porous and nanostructured nature of the films, accessible to the electrolyte.[8–12] To compare the two catalysts, the total amount of electroactive iridium (Figure 1C) is approximated as the total amount of redox state resulting from the 1st redox transition. This is done by integrating the 1st redox transition in Figures S2 and S7 for **IrO_x** and **$Ir_{Molecular}$** respectively.

Kinetics analysis

The kinetics decays were smoothed with a Savitzky-Golay filter using polynomial order 2 applied to a window of 50-100 points. To derive the lifetimes τ, the optical signal decays were normalized and fit with an initial slope linear regression between 0 and 25% intensity decay, following Equations S10-11:

Equation S10 $\Delta A = k \cdot t + c$

Equation S11 $\tau = 1/k$

Where ΔA is the experimental differential absorption, *t* is time, *k* and *c* are fit constants, and τ is the lifetime calculated from *k*.

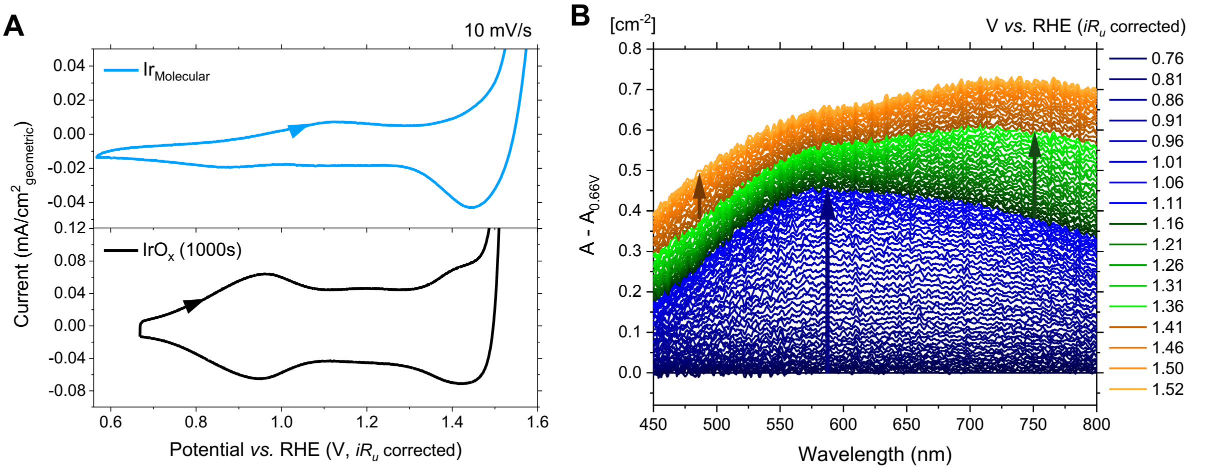

Figure S1. **(A)** Cyclic voltammetry of **$Ir_{Molecular}$** on mesoporous ITO (*top*) and **IrO_x** electrodeposited for 1000 s on FTO-glass (*bottom*). **(B)** Absorption changes at different potentials of the **IrO_x** electrode. The measurements were done in aqueous $HClO_4$ 0.1 M at pH 1.2. The cyclic voltammograms were recorded at a scan rate of 10 mV/s, starting at the open circuit potential of each electrode towards more oxidizing potentials as indicated by the arrow. The open circuit potential indicates the resting state of the catalyst, known to include Ir^{4+} in agreement with our assignment.[13]

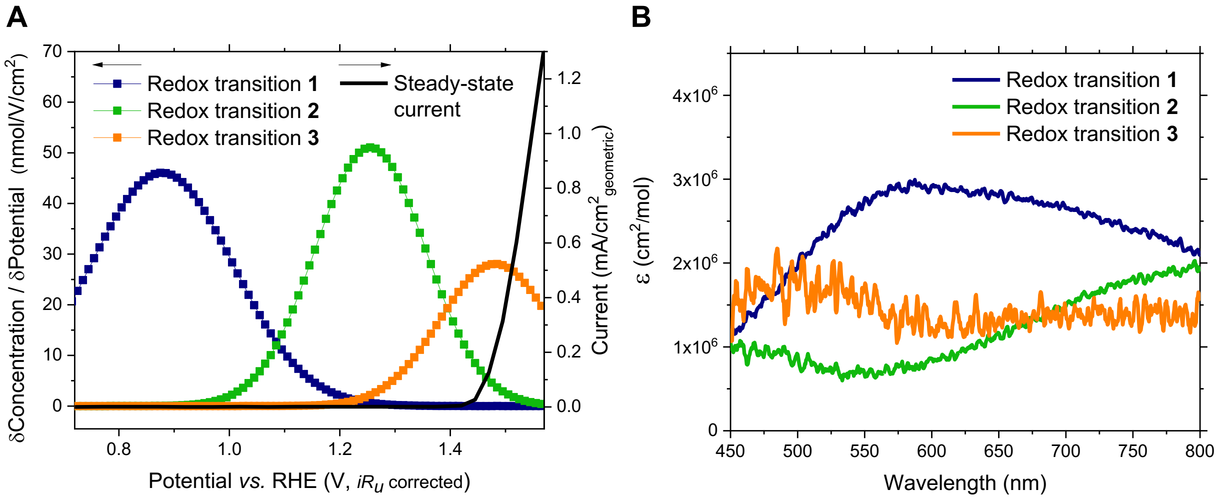

Figure S2. **(A)** Change in the concentration of redox states over potential, and **(B)** differential absorption coefficients (relative to 0.66 V *vs.* RHE) corresponding to the deconvolution and calibration of the three redox transitions fit to the spectroelectrochemical data of **IrO_x** in Figure S1B.

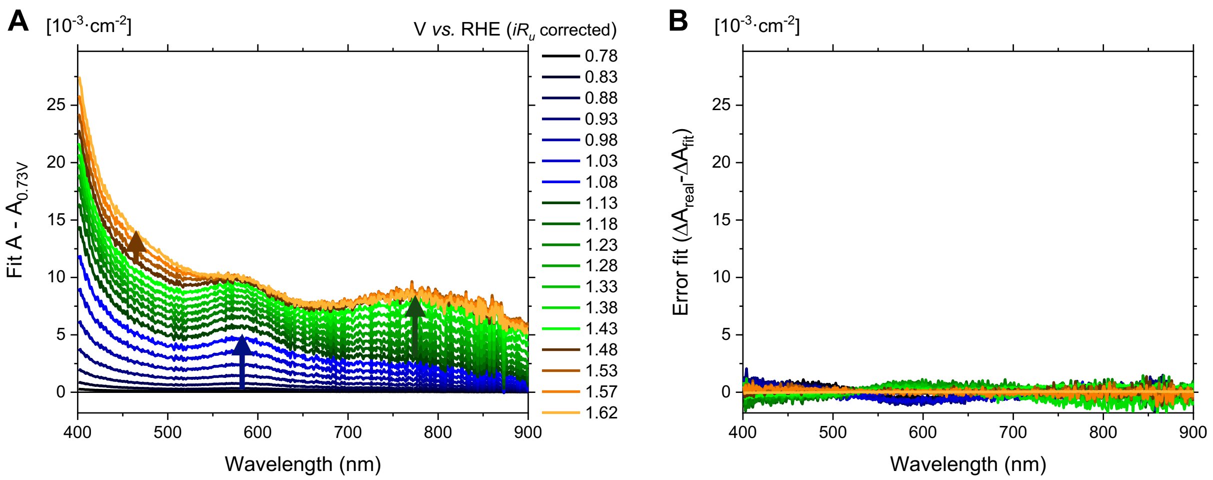

Figure S3. **(A)** Best fit of the spectroelectrochemical data of $\mathbf{Ir_{Molecular}}$ in Figure 2A generated with Equations S2-7. **(B)** Difference between the experimental data in Figure 2A and the model data in Figure S3A.

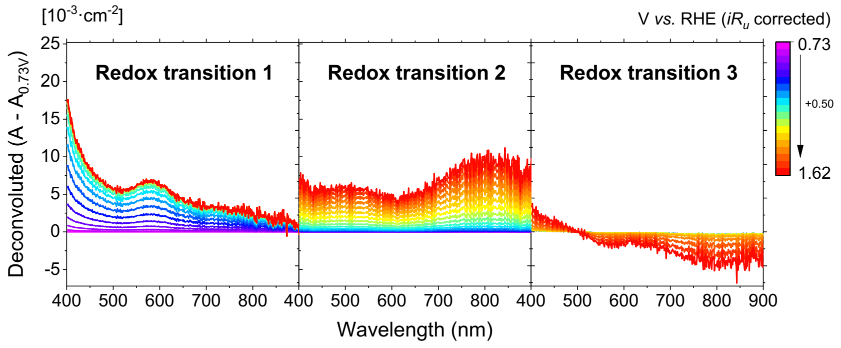

Figure S4. Absorption changes over potential associated to each redox transition of $\mathbf{Ir_{Molecular}}$ on mesoITO. This data was generated with Equations S2-7 and the sum of the three components at each potential yields the model data in Figure S3A.

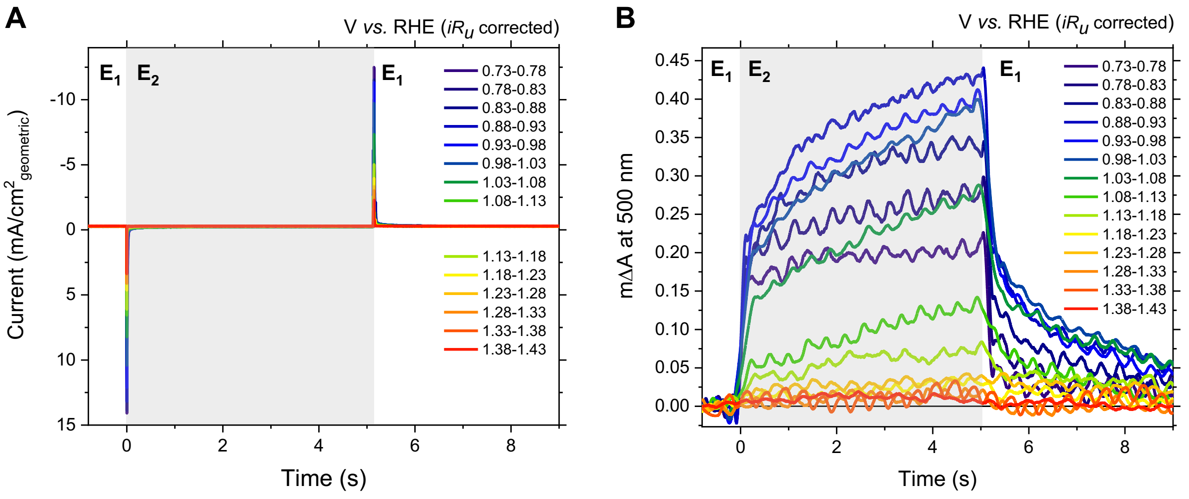

Figure S5. (A) Transient currents and **(B)** transient absorption changes measured in $\mathbf{Ir_{Molecular}}$ during potential steps between two different applied potentials in 0.1 M $HClO_4$ water at pH 1.2, as illustrated in Scheme S1.

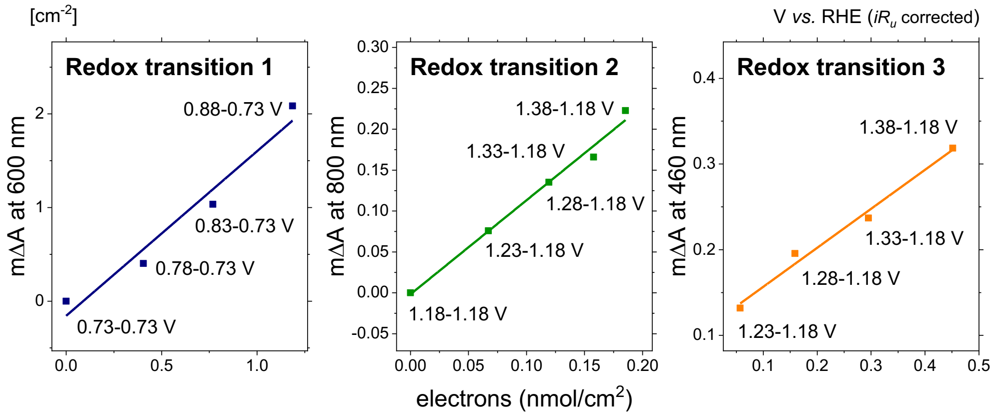

Figure S6. Dependency of the deconvoluted absorption changes on the extracted charge for each redox transition. The charges are calculated by integrating the reductive peak in Figure S5A and the deconvoluted absorption in Figure S4. The slope of the linear regression is the relative absorption coefficient of the new redox state formed at the corresponding redox transition. The label next to each data point indicates the potential interval where the absorption changes and the charges were measured.

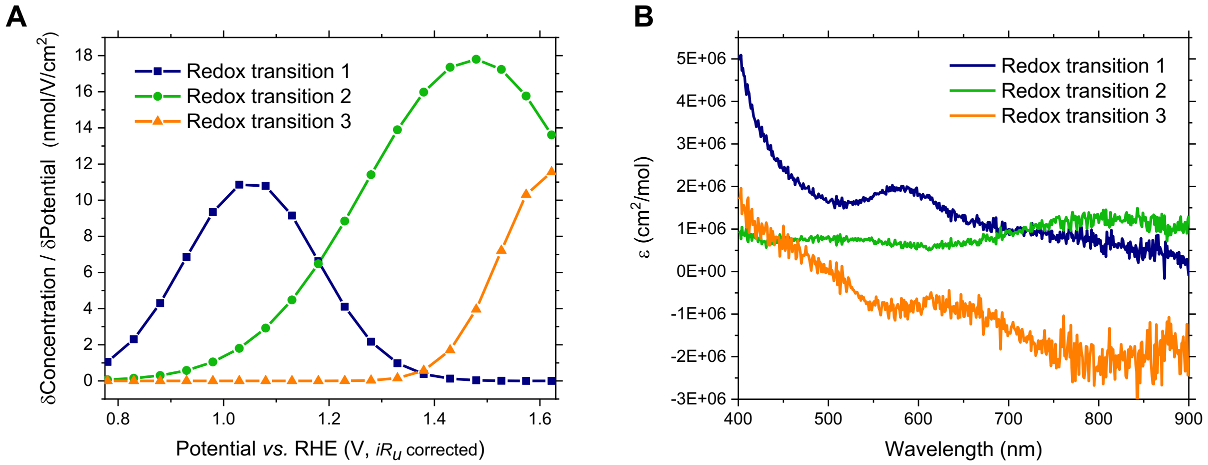

Figure S7. Deconvolution results of the spectroelectrochemical data of $Ir_{Molecular}$. (A) Change in the concentration of the three redox states formed at increasing potentials and (B) differential absorption coefficients of the corresponding redox transitions (relative to 0.73 V *vs.* RHE). These results are derived from the spectroelectrochemical experimental data in Figure 2A. Considering the concentration of states in the different redox transitions of $Ir_{Molecular}$ in Figure S7A, and the oxidation numbers of the iridium centers assigned according to the literature,[14,15] the 2nd redox transition of $Ir_{Molecular}$ most likely involves the oxidation of non-innocent coordinated ligands in addition to the iridium centers.

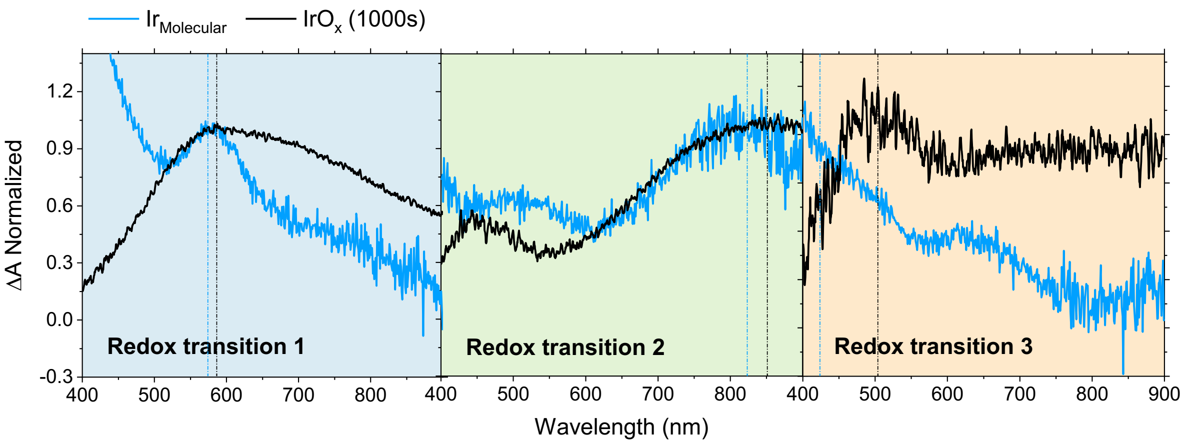

Figure S8. Differential absorption coefficient of the three redox transitions detected in IrO_x in aqueous $HClO_4$ 0.1 M at pH 1.2, shown also in Figure 2B right and relative to 0.73 V *vs.* RHE. Results calculated from fitting the spectroelectrochemistry data (Figures 2A and S3-7) and normalized at the maximum absorption of the corresponding characteristic band (at ~600, ~850 and ~500 nm for redox transitions 1, 2 and 3 respectively). The dashed lines show the maxima of these characteristic bands in *blue* for $Ir_{Molecular}$ and in *black* for IrO_x. Note that the band <500 nm in $Ir_{Molecular}$ could be the result of different bands overlapping, which would explain the larger spectral shift between $Ir_{Molecular}$ and IrO_x in redox transition 3 compared to the other two transitions.

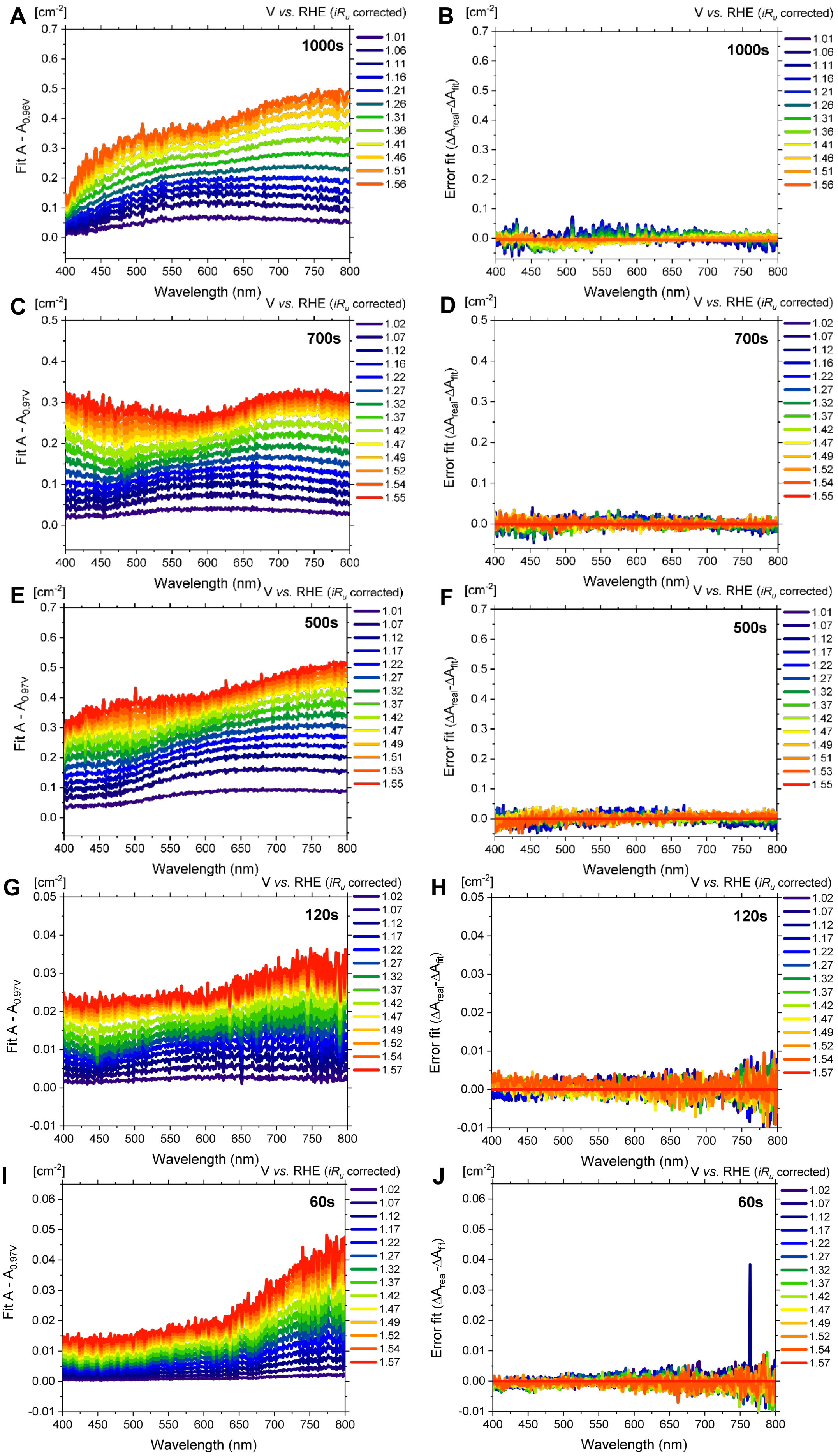

Figure S9. Fit spectroelectrochemistry data and calculation error of **IrO_x** electrodeposited for **(A-B)** 1000 s, **(C-D)** 700 s, **(E-F)** 500 s, **(G-H)** 120 s and **(I-J)** 60 s in 0.1 M $HClO_4$ water at pH 1.2. Model data generated with equations S2-7.

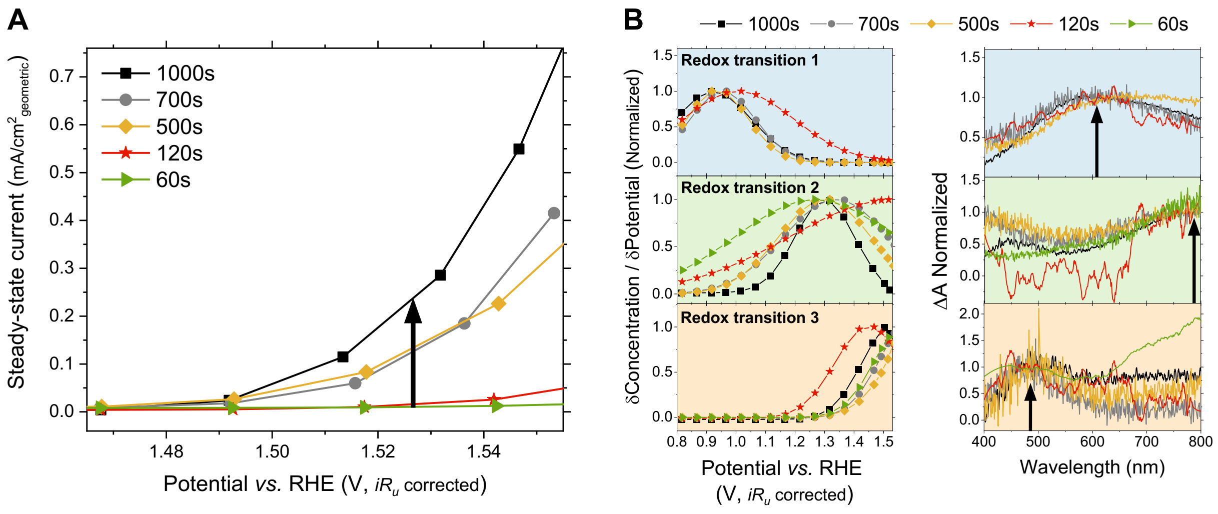

Figure S10. **(A)** Steady-state current and **(B)** normalized concentration changes and differential absorption coefficients obtained from spectroelectrochemical data of **IrO_x** samples electrodeposited for 1000s, 700s, 500s, 120s and 60s in 0.1 M $HClO_4$ water at pH 1.2.

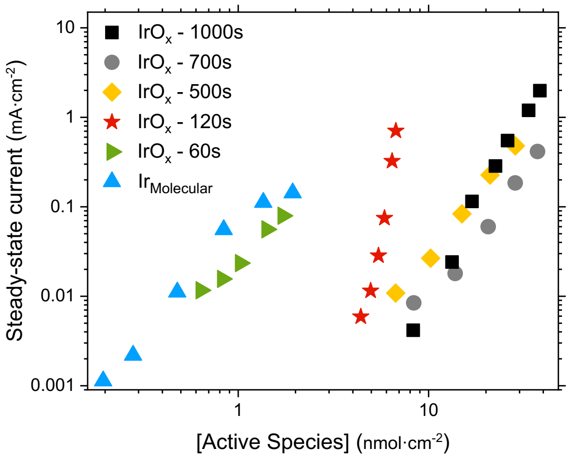

Figure S11. Steady-state current plotted against the concentration of active species in **IrO_x** samples with different electrodeposition times (1000s, 700s, 500s, 120s and 60s) and in **$Ir_{Molecular}$**. The concentration of active sites is derived from the deconvoluted and calibrated spectrooelectrochemical data (Figures 2B, S6-8) and the active state is assumed to be the redox state resulting from the 3rd redox transition.

Sample	*Molar ratio of redox states 1:2:3 at 1.57 V vs. RHE*
**IrO_x** 1000s	10 : 9 : 3
**IrO_x** 700s	6 : 10 : 3
**IrO_x** 500s	10 : 8 : 3
**IrO_x** 120s	10 : 10 : 3
**IrO_x** 60s	10 : 10 : 2 *
**$Ir_{Molecular}$**	6 : 10 : 1

Table S1. Ratio between the concentration of the redox states 1, 2 and 3 in each **IrO_x** and **$Ir_{Molecular}$** sample. The concentration in moles per cm^2 has been derived from the deconvolution of the spectroelectrochemistry data (Equations S2-7). (*) In **IrO_x** 60s, the first redox transition detected has been considered a combination of redox transitions 1 and 2 in the rest of the samples.

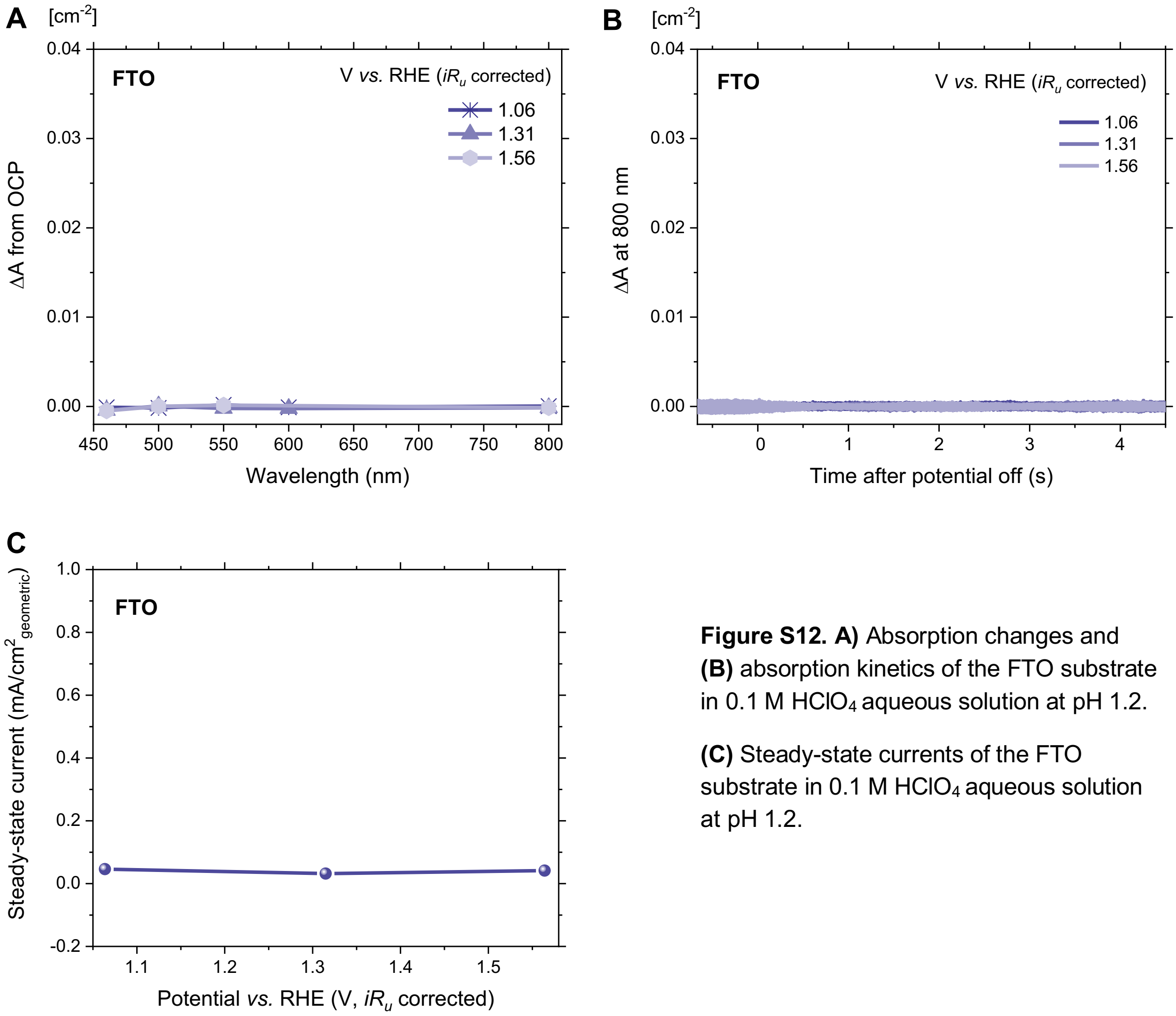

Figure S12. A) Absorption changes and **(B)** absorption kinetics of the FTO substrate in 0.1 M $HClO_4$ aqueous solution at pH 1.2.

(C) Steady-state currents of the FTO substrate in 0.1 M $HClO_4$ aqueous solution at pH 1.2.

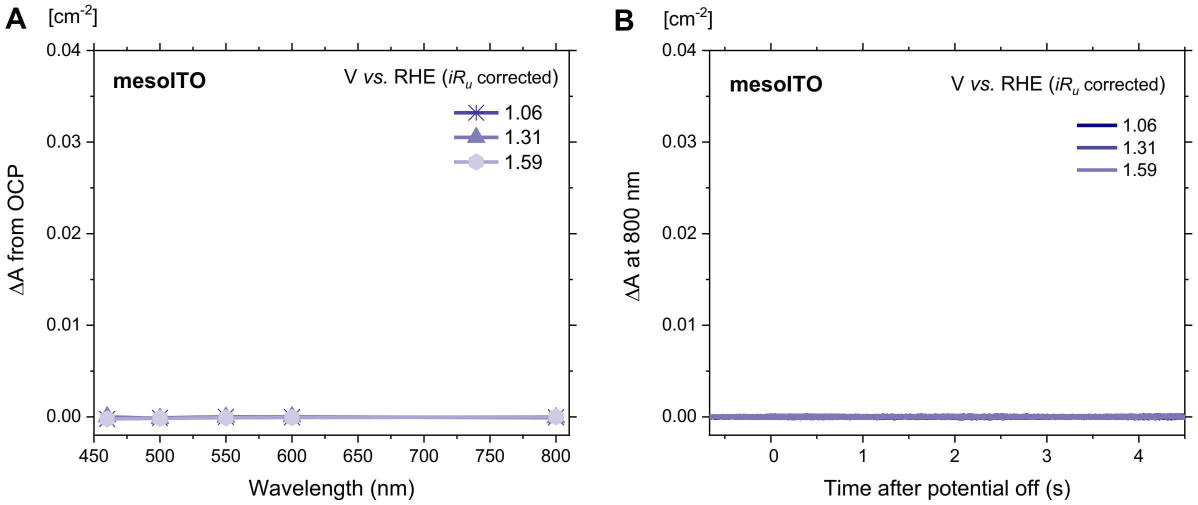

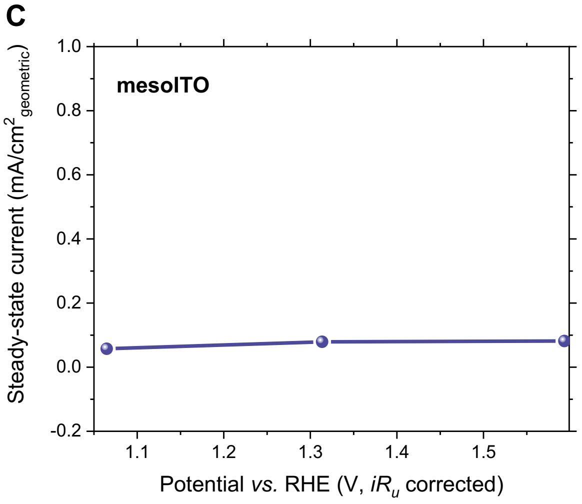

Figure S13. A) Absorption changes and **(B)** absorption kinetics of the mesoITO substrate in 0.1 M $HClO_4$ aqueous solution at pH 1.2.

(C) Steady-state currents of the mesoITO substrate in 0.1 M $HClO_4$ aqueous solution at pH 1.2.

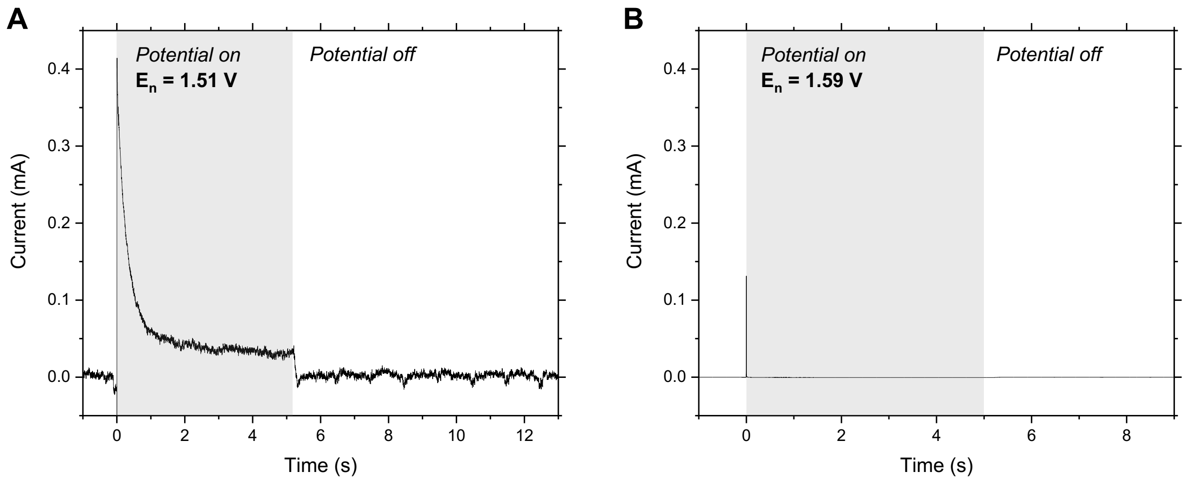

Figure S14. Transient current in **(A)** $\mathbf{IrO_x}$ and **(B)** $\mathbf{Ir_{Molecular}}$ when a potential is turned on for 5 s and off.

References

(1) Li, W.; He, D.; Sheehan, S. W.; He, Y.; Thorne, J. E.; Yao, X.; Brudvig, G. W.; Wang, D. Comparison of Heterogenized Molecular and Heterogeneous Oxide Catalysts for Photoelectrochemical Water Oxidation. *Energy & Environmental Science* **2016**, *9* (5), 1794–1802. https://doi.org/10.1039/c5ee03871e.

(2) Sheehan, S. W.; Thomsen, J. M.; Hintermair, U.; Crabtree, R. H.; Brudvig, G. W.; Schmuttenmaer, C. A. A Molecular Catalyst for Water Oxidation That Binds to Metal Oxide Surfaces. *Nat Commun* **2015**, *6*, 6469. https://doi.org/10.1038/ncomms7469.

(3) Li, W.; Sheehan, S. W.; He, D.; He, Y.; Yao, X.; Grimm, R. L.; Brudvig, G. W.; Wang, D. Hematite-Based Solar Water Splitting in Acidic Solutions: Functionalization by Mono- and Multilayers of Iridium Oxygen-Evolution Catalysts. *Angew Chem Int Ed Engl* **2015**, *54* (39), 11428–11432. https://doi.org/10.1002/anie.201504427.

(4) Thomsen, J. M.; Sheehan, S. W.; Hashmi, S. M.; Campos, J.; Hintermair, U.; Crabtree, R. H.; Brudvig, G. W. Electrochemical Activation of Cp* Iridium Complexes for Electrode-Driven Water-Oxidation Catalysis. *J Am Chem Soc* **2014**, *136* (39), 13826–13834. https://doi.org/10.1021/ja5068299.

(5) Hintermair, U.; Sheehan, S. W.; Parent, A. R.; Ess, D. H.; Richens, D. T.; Vaccaro, P. H.; Brudvig, G. W.; Crabtree, R. H. Precursor Transformation during Molecular Oxidation Catalysis with Organometallic Iridium Complexes. *J Am Chem Soc* **2013**, *135* (29), 10837–10851. https://doi.org/10.1021/ja4048762.

(6) Petit, M. A.; Plichon, V. Anodic Electrodeposition of Iridium Oxide Films. *Phys Chem Chem Phys* **1998**, *444*, 247.

(7) Zhang, Y.; Cao, M.; Lv, H.; Wei, J.; Gu, Y.; Liu, D.; Zhang, W.; Ryan, M. P.; Wu, X. Electrodeposited Nanometer-Size IrO2/Ti Electrodes with 0.3 mg IrO2 Cm−2 for Sludge Dewatering Electrolysers. *Electrochimica Acta* **2018**, *265*, 507–513. https://doi.org/10.1016/j.electacta.2018.01.190.

(8) Monk, P. M. S.; Mortimer, R. J.; Rosseinsky, D. R. Electrochromism and Electrochromic Devices. *Cambridge University Press* **2008**.

(9) Gottesfeld, S.; McIntyre, J. D. E.; Beni, G.; Shay, J. L. Electrochromism in Anodic Iridium Oxide Films. *Applied Physics Letters* **1978**, *33* (2), 208–210. https://doi.org/10.1063/1.90277.

(10) Monk, P. M. S.; Mortimer, R. J.; Rosseinsky, D. R. Electrochromism: Fundamental and Applications. *VCH* **1995**.

(11) Beni, G.; Rice, C. E.; Shay, J. L. Temperature Dependence of Electrochromic Processes in Iridium Oxide Displays. *J. Electrochem. Soc.* **1980**, *127*, 1342−1348.

(12) Schiavone, L. M.; Dautremont-Smith, W. C.; Beni, G.; Shay, J. L. Electrochromic Iridium Oxide Films Prepared by Reactive Sputtering. *Appl. Phys. Lett.* **1979**, *35* (823).

(13) Bartlett, S. A.; Sackville, E. V.; Gibson, E. K.; Celorrio, V.; Wells, P. P.; Nachtegaal, M.; Sheehan, S. W.; Hintermair, U. Evidence for Tetranuclear Bis-μ-Oxo Cubane Species in Molecular Iridium-Based Water Oxidation Catalysts from XAS Analysis. *Chem. Commun.* **2019**, *55* (54), 7832–7835. https://doi.org/10.1039/C9CC02088H.

(14) Sharninghausen, L. S.; Bhushan Sinha, S.; Shopov, D. Y.; Mercado, B. Q.; Balcells, D.; Brudvig, G. W.; Crabtree, R. H. Synthesis and Characterization of Iridium (V) Coordination Complexes With an N,O-Donor Organic Ligand. *Angew. Chem.* **2017**, *129*, 13227–13231.

(15) Sinha, S. B.; Shopov, D. Y.; Sharninghausen, L. S.; Stein, C. J.; Mercado, B. Q.; Balcells, D.; Pedersen, T. B.; Reiher, M.; Brudvig, G. W.; Crabtree, R. H. Redox Activity of Oxo-Bridged Iridium Dimers in an N,O-Donor Environment: Characterization of Remarkably Stable Ir(IV,V) Complexes. *J Am Chem Soc* **2017**, *139* (28), 9672–9683. https://doi.org/10.1021/jacs.7b04874.
